# Outcomes of Spinal Cord Infarction with Thrombolysis: A Nationwide Analysis

**DOI:** 10.1007/s12028-025-02251-y

**Published:** 2025-04-03

**Authors:** Ali Al-Salahat, Danielle B. Dilsaver, Ali Bin Abdul Jabbar, Saif Bawaneh, Abhishek Singh, Evanthia Bernitsas

**Affiliations:** 1 Neurology Department, Creighton University, Omaha, NE USA; 2 Department of Clinical Research and Public Health, Creighton University, Omaha, NE USA; 3 Department of Medicine, Creighton University, Omaha, NE USA

## Introduction

Spinal cord infarction (SCI) accounts only for 0.3% to 1% of all types of stroke [1, 2]. Clinical presentation of SCI consists of an acute myelopathy, accompanied by sphincteric and autonomic dysfunction, and back pain [2, 3]. Data on the management of SCI are limited, lacking large randomized controlled trials. Studies that examined outcomes of SCI were limited in sample size but showed that age and comorbidity burden were important prognostic indicators [4, 5]. Given the rarity of SCI, standard treatments for ischemic stroke, such as thrombolysis, have not been previously studied. Only a few case reports and series suggested potential beneficial effects of thrombolysis in SCI [6, 7]. In this study, we aim to assess the outcomes of SCI with thrombolysis in a large sample representative of the United States using the Nationwide Readmissions Database (NRD).

## Methods

This study followed the STROBE reporting guidelines [8]. Hospitalization data were abstracted from the 2016–2021 NRD, which is an all-payer, publicly available administrative inpatient care database sponsored by the Agency for Healthcare Research and Quality and estimates approximately 35 million yearly discharges in the United States [9]. We identified hospitalization of patients with SCI using *International Classification of Diseases, Tenth Revision* (ICD-10) codes (G95.11). Hospitalizations were excluded if the patient was younger than 18 years. The Institutional Review Board of Creighton University acknowledged this study as not human subjects research (InfoEd record number: 2004608). The advantage of using the NRD as the data source for our study is that readmission rates are considered good indicators of long-term morbidity in ischemic stroke. Additionally, it allows us to capture a large number of hospitalizations when it pertains to rare conditions and exposures, such as SCI and thrombolysis.

Outcomes were inpatient mortality, length of stay (LOS), inflation-adjusted cost [10], routine discharge (i.e., home/self-care), and 30- and 90-day all-cause readmissions. We stratified these outcomes based on receipt of thrombolysis (ICD-10-CM code Z92.82, ICD-10-PCS codes 3E03017 and 3E03317). To allow for complete postdischarge follow-up for readmission analyses, if a patient died, the hospitalization was excluded from the 30- and 90-day readmission outcome analyses. Relatedly, the NRD does not track patients year to year; index hospitalizations in which the patient was discharged in December were excluded for the 30-day readmission analysis, and index hospitalizations in which the patient was discharge in October, November, or December were excluded for the 90-day readmission analysis. Additional sensitivity analysis was performed to compare outcomes between primary and secondary SCI.

We extracted descriptive data, including age, biological sex, comorbidity burden, and National Institutes of Health Stroke Scale (NIHSS) score (Supplemental Table I). Comorbidity burden was quantified using the Charlson Comorbidity Index (CCI), a score developed for health care research to assess patient risk [11]. We also identified whether the patient underwent an aortic repair during the hospitalization using ICD-10 procedures codes (open approach: 02QX0ZZ, 02QW0ZZ, 04Q00ZZ; percutaneous approach: 02QX3ZZ, 02QW3ZZ, 04Q03ZZ).

Continuous descriptive data were presented as median and interquartile range and categorical descriptive data were presented as weighted frequency and percentage. To assess whether inpatient mortality, routine discharge, and 30- and 90-day all-cause readmissions differed by thrombolysis, we estimated logistic regression models. Likewise, we estimated log-normal models to evaluate whether LOS and hospital cost differed by thrombolysis. Multivariable regression models that controlled for age and comorbidity burden (CCI) were estimated. The NRD sampling design was accounted for in all analyses; a two-tailed *p* value < 0.05 indicated statistical significance.

## Results

Over the study period, there were an estimated 12,548 (95% confidence interval [CI] 12,085–13,010) hospitalized patients with an SCI diagnosis. Table 1 shows the baseline characteristics stratified by receipt of thrombolysis. Common comorbidities associated with SCI were peripheral arterial disease (PAD) (37.58%, weighted *n* = 4,715), chronic obstructive pulmonary disease (23.00%, weighted *n* = 2,886), congestive heart failure (22.22%, weighted *n* = 2,788), and AIDS (21.46%, weighted *n* = 2,693).

**Table 1:** Baseline characteristics and overall outcomes in hospitalizations of patients with SCI, stratified by receipt of thrombolysis

	Overall	Thrombolysis	
		No	Yes
Age, median (IQR), years	63.43 (52.60–73.06)	63.41 (52.58–73.02)	64.90 (55.55–76.99)
Charlson Comorbidity Index, median (IQR)	3.44 (1.80–7.08)	3.45 (1.79–7.10)	2.95 (2.10–5.63)
Sex, %			
Male	58.79	58.9	49.52
Female	41.21	41.1	50.48
NIHSS, %^*a*^			
0–9	66.37	67.20	59.87
10 +	33.63	32.80	–^b^
Reasons for hospitalization, %			
Primary SCI diagnosis	21.43	21.26	35.76
Secondary SCI diagnosis	78.57	78.74	64.24
Index hospitalization outcomes			
Inpatient morality, %	14.66	14.6	19.45
Length of stay, days	13.09	13.12	10.83
Cost, $	53,531	53,551	51,869
Routine discharge, %	19.94	20.04	–^b^
All-cause readmission outcomes, %			
30-day	21.08	22.44	12.93
90-day	34.01	34.21	15.11

Categorical characteristics are presented as percentages

IQR, interquartile range, NIHSS, National Institutes of Health Stroke Scale, SCI, spinal cord infarction

^a^The percentage is out of hospitalizations with a provided NIHSS score (weighted *n* = 464)

^b^The results could not be presented per the Nationwide Readmissions Database Data Use Agreement, as there were 10 or less hospitalizations. Routine discharge is defined as discharge home or to self-care

Hospitalized patients with SCI had an inpatient morality rate of 14.66% (95% CI 13.74–15.59). The average LOS was 13.09 days (95% CI 12.61–13.59), and the average cost was $53,531 (95% CI $51,615–$55,518). Relatedly, 16.86% (95% CI 15.78–17.94%) of hospitalizations of patients with SCI resulted in a routine discharge. For all-cause readmissions, 21.08% (95% CI 19.86–22.30%) and 34.01% (95% CI 32.36–35.65%) of patients with SCI were readmitted within 30 and 90 days, respectively.

**Fig. 1:**
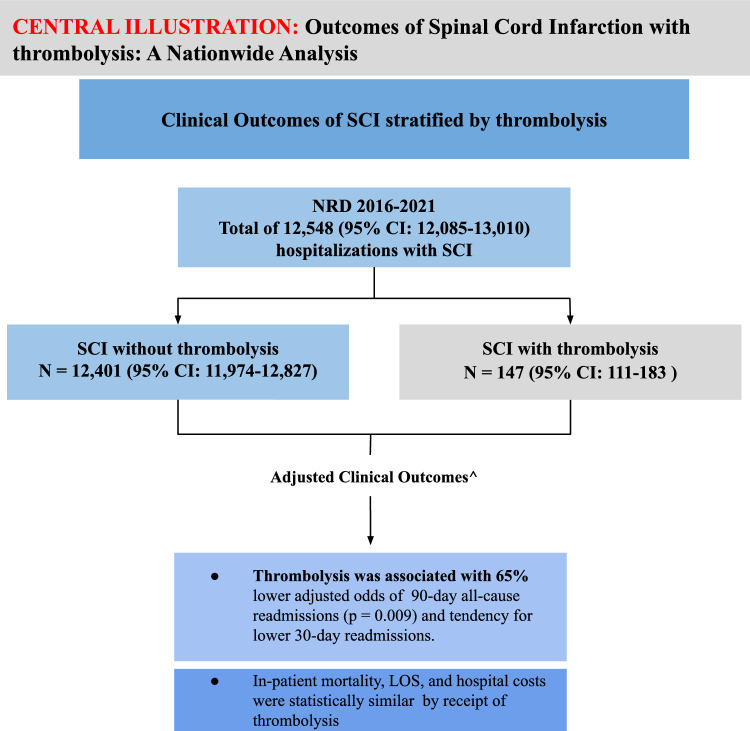
Central illustration summarizing hospitalizations selection and main results. An ^ indicates outcomes were adjusted for age and comorbidity burden. CI confidence interval, LOS length of stay, NRD Nationwide Readmissions Database, SCI spinal cord infarction

Notably, 1.17% (weighted *n* = 147, 95% CI 111–183) of hospitalized patients with SCI received thrombolysis (Fig. 1). Outcomes by whether patients with SCI received thrombolysis are presented in Table 2. Inpatient mortality, LOS, routine discharge, and all-cause 30-day readmission were statistically similar between hospitalization with thrombolysis and hospitalization without thrombolysis. After adjusting for age and CCI, the adjusted odds of 90-day all-cause readmissions were 65% lower for index hospitalizations of patients receiving thrombolysis compared to no thrombolysis (adjusted odds ratio 0.35, 95% CI 0.16–0.77, *p* = 0.009; Table 2). The comparison between primary and secondary SCI is detailed in Supplemental Table II.

**Table 2:** Hospitalizations outcomes by whether patients with SCI received thrombolysis

	Unadjusted				Adjusted^a^	
	Thrombolysis: no	Thrombolysis: yes	Odds ratio (95% CI)	*p* value	Odds ratio (95% CI)	*p* value
Index hospitalization						
Inpatient morality	14.6%	19.45%	1.41 (0.71–2.83)	0.33	1.46 (0.77–2.77)	0.249
LOS	13.12 days	10.83 days	0.83 (0.61–1.11)	0.207	0.86 (0.65–1.14)	0.294
Cost	$53,551	$51,869	0.97 (0.78–1.18)	0.747	1.01 (0.84–1.20)	0.941
Routine discharge	20.04%	–^b^	–^b^	–^b^	–^b^	–^b^
All-cause readmission						
30-day	22.44%	12.93%	0.55 (0.27–1.15)	0.114	0.58 (0.28–1.20)	0.142
90-day	34.21%	15.11%	0.34 (0.16–0.75)	0.008	0.35 (0.16–0.77)	0.009

CI, confidence interval, LOS, length of stay. SCI, spinal cord infarction

^a^Outcomes were adjusted for age and comorbidity burden

^b^The results could not be presented per the Nationwide Readmissions Database Data Use Agreement, as there were 10 or less hospitalizations. Routine discharge is defined as discharge home or to self-care

## Discussion

This study showed several notable findings. First, there were an estimated 12,548 hospitalizations of patients with an SCI diagnosis in the United States from 2016 to 2021, with patients having a median age of 63.43 years. Men were affected by SCI more than women (58.79% vs. 41.21%). The most common comorbidities associated with SCI were PAD, chronic obstructive pulmonary disease, congestive heart failure, and AIDS. Only an estimated 68 hospitalized patients with SCI underwent an open aortic repair, and no hospitalizations were found to occur with percutaneous aortic repair. Second, 14.66% of hospitalizations of patients with SCI resulted in inpatient death, whereas 16.86% resulted in routine discharge. On average, patients with SCI were hospitalized for 13.09 days, with an average cost of $53,531. Readmissions after SCI occurred in 21.08% at 30 days and 34.01% at 90 days after discharge. Lastly, around 147 (1.17%) hospitalizations of patients with SCI were associated with thrombolysis. After adjusting for age and comorbidity burden, thrombolysis was associated with significantly lower all-cause 90-day readmissions, with a tendency toward lower 30-day readmissions. Inpatient mortality, LOS, and hospital costs were similar by receipt of thrombolysis.

Given the rarity of SCI, previous studies have been limited in numbers [2, 3]. Our study provides descriptive demographic and clinical outcomes in hospitalizations of patients with SCI. Consistent with our study, previous studies demonstrate an average age at onset between 57 and 72 years, with a predominance in men [1, 5, 12]. PAD was the most common comorbidity associated with SCI. Data exist to support the association between PAD and aortic pathology, such as abdominal aortic aneurysm, which increases the risk for SCI [13]. Other associated comorbidities were chronic obstructive pulmonary disease, serving as a surrogate of smoking risk, and congestive heart failure, which indicates an overall cardiovascular risk profile. Interestingly, AIDS was a relatively frequent comorbidity; 21.46% of hospitalized patients with SCI had AIDS. Anecdotal evidence exists to suggest an association between opportunistic infections, such as Varicella zoster virus, and vasculitis leading to SCI [14]. This finding warrants further investigation into the association between AIDS and SCI. Notably, none of the hospitalized patients with SCI had an endovascular aortic surgical repair, and only 68 had open aortic surgical repair. Recent evidence shows that there has been a significant up-trend in endovascular aortic repairs, with open aortic repairs significantly decreasing over the past two decades [15]. A meta-analysis showed that endovascular aortic repairs carry significantly lower risk for SCI than open aortic repairs, and our findings highlight this as well [16].

Additionally, we described overall outcomes and health care use metrics for hospitalizations of patients with SCI. The literature lacks such data on SCI given limited sample sizes in previous studies. Health care use metrics, such as LOS, hospital costs, all-cause readmissions, and routine discharge, provide important data for neurologists, rehabilitation physicians, health care administrators, and economists. Our findings highlight the overall burden of SCI in terms of inpatient mortality, LOS, and hospital costs. These data may also help guide the inpatient management and postdischarge care of patients with SCI.

This study found an association between thrombolysis and lower readmissions after SCI. Despite the limited number of patients who received thrombolysis compared to the total cohort of patients with SCI, this association between thrombolysis and SCI hospitalization outcomes has not been previously studied. Moreover, the association remained significant after adjusting for age and comorbidity burden. Studies have previously shown that recovery after SCI can take several months [2, 4]. It is, therefore, plausible that thrombolysis may help in the long-term recovery after SCI. This can also explain the findings of a tendency toward lower 30-day all-cause readmissions and statistical difference between those with and without thrombolysis for 90-day all-cause readmissions. Relatedly, a study that analyzed the NRD for cerebral ischemic stroke found significantly lower odds of 30-day readmissions with recanalization therapy, which included thrombolysis and thrombectomy [17].

This study has some limitations. The NRD is an administrative database reliant on billing codes, and nonreimbursable codes (e.g., NIHSS diagnosis codes) may be underreported. The NRD does not provide information on long-term functional outcomes. Despite being one of the largest studies investigating SCI hospitalization outcomes, we were still limited by sample size and a low number of patients receiving thrombolysis.

Overall, our results provide insights into outcomes and health care use metrics relating to SCI. Additionally, the findings uncover the association between thrombolysis and better long-term outcomes in SCI. This paves the way for further investigation into the role of thrombolysis in SCI. Future observational studies with granular-level data may help establish the benefit and risk of thrombolysis in SCI.

## Electronic supplementary material

Below is the link to the electronic supplementary material.

Supplementary file 1 (DOCX 17.6 kb)

Supplementary file 1 (DOCX 33.9 kb)

## Data Availability

The data used in this study are publicly available.
