# Supplementary material for: Outcomes of Spinal Cord Infarction with Thrombolysis: A Nationwide Analysis
Source: Neurocrit Care. 2025 Apr 3;42(3):1102–6. doi: 10.1007/s12028-025-02251-y (PMC12137411; doi:10.1007/s12028-025-02251-y)
Supplement: Supplementary file 1 — (DOCX 17.6 kb) [file 12028_2025_2251_MOESM1_ESM.docx]

| NIHSS  ( National Institute of Health Stroke Scale) | ICD-10-CM |
| --- | --- |
| NIHSS score 0-9 | R29.70 |
| NIHSS score 10-19 | R29.71 |
| NIHSS score 20-29 | R29.72 |
| NIHSS score 30-39 | R29.73 |
| NIHSS score 40-42 | R29.74 |

**Supplemental Table I.** *International Classification of Diseases, Tenth Revision* clinical modification (ICD-10-CM) codes used to identify National Institute of Health Stroke Scale.

| **Supplemental Table II.** Hospitalizations outcomes by whether spinal cord infarction was the primary diagnosis. | | | | |
| --- | --- | --- | --- | --- |
|  | Primary SCI Diagnosis | Secondary SCI Diagnosis | Ratio (95% CI) | p |
| Index hospitalization |  |  |  |  |
| Inpatient morality, % | 3.82 | 17.62 | 0.19 (0.14, 0.25) | < 0.001 |
| Length of stay, days | 9.17 | 14.43 | 0.64 (0.57, 0.71) | < 0.001 |
| Cost, $ | 28,138 | 63,763 | 0.44 (0.41, 0.47) | < 0.001 |
| Routine discharge, % | 24.55 | 18.47 | 1.44 (1.22, 1.69) | < 0.001 |
| All-cause Readmission |  |  |  |  |
| 30-day, % | 15.10 | 23.00 | 0.60 (0.50, 0.71) | < 0.001 |
| 90-day, % | 27.30 | 36.23 | 0.66 (0.56, 0.78) | < 0.001 |
