# Supplementary material for: Outcomes of Spinal Cord Infarction with Thrombolysis: A Nationwide Analysis
Source: Neurocrit Care. 2025 Apr 3;42(3):1102–6. doi: 10.1007/s12028-025-02251-y (PMC12137411; doi:10.1007/s12028-025-02251-y)
Supplement: Supplementary file 2 — Supplementary file 1 (DOCX 33.9 kb) [file 12028_2025_2251_MOESM2_ESM.docx]

STROBE Statement—checklist of items that should be included in reports of observational studies

|  | Item No | Recommendation | Page/line No |
| --- | --- | --- | --- |
| **Title and abstract** | 1 | (*a*) Indicate the study’s design with a commonly used term in the title or the abstract | Lines 1-2 |
|  |  | (*b*) Provide in the abstract an informative and balanced summary of what was done and what was found | Page 1,2 |
| Introduction | | | |
| Background/rationale | 2 | Explain the scientific background and rationale for the investigation being reported | Lines 72-104 |
| Objectives | 3 | State specific objectives, including any prespecified hypotheses | Lines 99-104 |
| Methods | | | |
| Study design | 4 | Present key elements of study design early in the paper | Lines 108-113 |
| Setting | 5 | Describe the setting, locations, and relevant dates, including periods of recruitment, exposure, follow-up, and data collection | Lines 108-113 |
| Participants | 6 | (*a*) *Cohort study*—Give the eligibility criteria, and the sources and methods of selection of participants. Describe methods of follow-up  *Case-control study*—Give the eligibility criteria, and the sources and methods of case ascertainment and control selection. Give the rationale for the choice of cases and controls  *Cross-sectional study*—Give the eligibility criteria, and the sources and methods of selection of participants | Lines 108-115 |
|  |  | (*b*) *Cohort study*—For matched studies, give matching criteria and number of exposed and unexposed  *Case-control study*—For matched studies, give matching criteria and the number of controls per case | Na |
| Variables | 7 | Clearly define all outcomes, exposures, predictors, potential confounders, and effect modifiers. Give diagnostic criteria, if applicable | Lines 117-136 |
| Data sources/ measurement | 8* | For each variable of interest, give sources of data and details of methods of assessment (measurement). Describe comparability of assessment methods if there is more than one group | *Page 5-6* |
| Bias | 9 | Describe any efforts to address potential sources of bias | Page 5-6 |
| Study size | 10 | Explain how the study size was arrived at | Lines 108-109 |
| Quantitative variables | 11 | Explain how quantitative variables were handled in the analyses. If applicable, describe which groupings were chosen and why | Lines 138-152 |
| Statistical methods | 12 | (*a*) Describe all statistical methods, including those used to control for confounding | Lines 154-167 |
|  |  | (*b*) Describe any methods used to examine subgroups and interactions | Lines 154-167 |
|  |  | (*c*) Explain how missing data were addressed | Lines 131-136 |
|  |  | (*d*) *Cohort study*—If applicable, explain how loss to follow-up was addressed  *Case-control study*—If applicable, explain how matching of cases and controls was addressed  *Cross-sectional study*—If applicable, describe analytical methods taking account of sampling strategy | Lines 131-136 |
|  |  | (*e*) Describe any sensitivity analyses | na |

Continued on next page

| Results | | | |
| --- | --- | --- | --- |
| Participants | 13* | (a) Report numbers of individuals at each stage of study—eg numbers potentially eligible, examined for eligibility, confirmed eligible, included in the study, completing follow-up, and analysed | Lines 170-174 |
|  |  | (b) Give reasons for non-participation at each stage | Na |
|  |  | (c) Consider use of a flow diagram | Na |
| Descriptive data | 14* | (a) Give characteristics of study participants (eg demographic, clinical, social) and information on exposures and potential confounders | Page 21 |
|  |  | (b) Indicate number of participants with missing data for each variable of interest | Na |
|  |  | (c) *Cohort study*—Summarise follow-up time (eg, average and total amount) | Lines 191-193 |
| Outcome data | 15* | *Cohort study*—Report numbers of outcome events or summary measures over time | *Lines 184-193* |
|  |  | *Case-control study—*Report numbers in each exposure category, or summary measures of exposure | *Na* |
|  |  | *Cross-sectional study—*Report numbers of outcome events or summary measures | *Na* |
| Main results | 16 | (*a*) Give unadjusted estimates and, if applicable, confounder-adjusted estimates and their precision (eg, 95% confidence interval). Make clear which confounders were adjusted for and why they were included | Lines 183-235 |
|  |  | (*b*) Report category boundaries when continuous variables were categorized | Na |
|  |  | (*c*) If relevant, consider translating estimates of relative risk into absolute risk for a meaningful time period | Na |
| Other analyses | 17 | Report other analyses done—eg analyses of subgroups and interactions, and sensitivity analyses | Lines 200-235 |
| Discussion | | | |
| Key results | 18 | Summarise key results with reference to study objectives | 309-314 |
| Limitations | 19 | Discuss limitations of the study, taking into account sources of potential bias or imprecision. Discuss both direction and magnitude of any potential bias | 302-306 |
| Interpretation | 20 | Give a cautious overall interpretation of results considering objectives, limitations, multiplicity of analyses, results from similar studies, and other relevant evidence | Lines 238-306 |
| Generalisability | 21 | Discuss the generalisability (external validity) of the study results | Lines302-304 |
| Other information | | | |
| Funding | 22 | Give the source of funding and the role of the funders for the present study and, if applicable, for the original study on which the present article is based | Lines 319-320 |

*Give information separately for cases and controls in case-control studies and, if applicable, for exposed and unexposed groups in cohort and cross-sectional studies.

**Note:** An Explanation and Elaboration article discusses each checklist item and gives methodological background and published examples of transparent reporting. The STROBE checklist is best used in conjunction with this article (freely available on the Web sites of PLoS Medicine at http://www.plosmedicine.org/, Annals of Internal Medicine at http://www.annals.org/, and Epidemiology at http://www.epidem.com/). Information on the STROBE Initiative is available at www.strobe-statement.org.
